# The Feasibility and Acceptability of a Stand-Alone Virtual Reality Headset on Perceived Pain and Anxiety During Bone Marrow Biopsies: Mixed Methods Pilot Study

**DOI:** 10.2196/65324

**Published:** 2025-09-08

**Authors:** Ajay Mittal, Suhaiba Huq, Jonathan Wakim, Kevin Kapadia, Tung Wynn

**Affiliations:** 1 Department of Medicine, University of Florida College of Medicine, 1600 SW Archer Rd, Gainesville, FL, 32610, United States, 1 3524259602; 2 BayCare Health System/Northwestern University Feinberg School of Medicine, Clearwater, FL, United States; 3 Department of Medicine, University of Pennsylvania Perelman School of Medicine, Philadelphia, PA, United States; 4 Department of Medicine, University of Southern California, Los Angeles, CA, United States

**Keywords:** virtual reality, bone marrow biopsy, oncology, pharmacologic pain management, pain management

## Abstract

**Background:**

Virtual reality (VR) is an emerging technology that provides an immersive user experience and has the ability to distract patients from the negative or painful experiences commonly associated with medical procedures. Bone marrow biopsies are medical procedures where a needle is inserted into the bone and a syringe is used to withdraw the liquid bone marrow. They are performed to diagnose and monitor disorders affecting the blood, often as part of care for hematology and patients with cancer.

**Objective:**

The purpose of this pilot study is to assess the feasibility of VR as an adjunctive therapy to alleviate the perception of pain and anxiety in patients receiving bone marrow biopsies.

**Methods:**

This pilot study enrolled 60 adult participants receiving a bone marrow biopsy to assess the acceptability and feasibility of VR to impact reported pain and anxiety levels compared to the participants’ baseline measurements preoperatively. They were randomly assigned to “control”/non-VR intervention (n=30) and “experimental”/VR groups (n=30). The “experimental”/VR group used the Meta Quest 2 headset (Meta) with original VR content developed for this study. Participants completed a survey adapted from a standardized verbal numerical rating scale to rate their pain and anxiety levels before and after the bone marrow biopsy. Measurements such as procedure length, patient vitals, and experience were also gathered from both study groups.

**Results:**

Results indicated that participants had no significant differences in their heart rate, respiration rate, and blood oxygen saturation levels between the 2 groups. Participants in the VR group (n=30) had a significantly shorter procedure length than the control group (n=30) with a 25% time reduction (*P*=.02). Participants in the VR group (mean 4.29, SD 1.19) were significantly more likely to rate the distraction as effective (*P*<.001) and report they would repeat the procedure (mean 4.32, SD 1.05; *P*<.001). Finally, participants in the VR group (mean 2.13, SD 1.26) had significantly lower levels of anxiety during the procedure (*P*<.001) and felt significantly more comfortable after the procedure (mean 4.45, SD 1.12; *P*<.001).

**Conclusions:**

This investigation encourages the acceptability of using VR intervention for patients undergoing bone marrow biopsies. Further, the length of procedures was found to be shorter when compared to the control group, supporting the feasibility of the technology for clinical management. These novel interventions can provide distraction-based therapy that is noninferior to the standard of care and provide enjoyable user experiences that reduce the perceived pain and anxiety of nonsedated medical procedures.

## Introduction

Standard pain management protocols in adult medicine settings, hospitals, or clinics, typically rely on the use of pharmacotherapies such as acetaminophen, nonsteroidal anti-inflammatory drugs, analgesics, and opioids to alleviate acute pain [1]. Although these forms of treatment can be effective, there are growing concerns surrounding the potential health risks associated with the use of certain pharmacotherapies, such as opioid addiction, across patient populations [2]. As a result, there is a growing interest in reducing the use of pharmacotherapy for the treatment of acute pain in favor of nonpharmacologic options [3]. However, there are few alternative options for providing nonpharmacologic therapy in adults, which often results in inadequate pain control [4]. There is a need to find feasible and acceptable adjunct forms of pain management and anxiety reduction in a hospital or clinic setting.

Distraction techniques used by hospital staff help patients cope with injuries, hospitalization, or illness and differ based on each patient’s needs and preferences [5]. Some common techniques used to distract from chronic pain include controlled breathing, guided imagery, and relaxation [6]. Passive techniques such as auditory distraction and television have been used to distract from acute pain resulting from routine procedures [7]. Studies for each of these different techniques over the years found some positive, but mostly mixed results [8]. Thus, there is still limited evidence demonstrating the effectiveness of distraction [9]. Moreover, there is no conclusive study suggesting that one technique supersedes others since each patient has different preferences, medical situations, behavioral needs, and developmental needs.

Virtual reality (VR) is an emerging technology that provides an immersive user experience [10]. These immersive VR experiences show promise as a tool that can reduce perceived pain and anxiety related to acute pain in emergency rooms or other clinical settings by lessening the vividness of memories [11]. One study has even observed that immersive VR was more effective than standard care in ameliorating pain among pediatric patients undergoing venipunctures [12]. It is theorized that immersive VR draws the patient’s attention away from aversive, or painful, stimuli by keeping their focus on something more engaging [13]. A study published in 2023 by Alaniz et al [14] found VR to be an underused intraoperative tool that enhances the overall patient experience in the emergency room. Furthermore, another study by Sabinash [15] reported positive findings with this technology, which are tempered by the limitations of current research, but VR still holds great potential. The stand-alone headset’s hardware offers an affordable alternative that can potentially provide a nonpharmacologic treatment for acute pain management and anxiety reduction [16]. A study published in Virtual Worlds found that it is likely to see the widespread implementation of VR in health care in the coming decade [17]. With recent advancements, VR technology is used in numerous settings, but limited research has been done thus far to show how effective it is [18].

The purpose of this pilot study is to evaluate the feasibility and acceptability of using VR adjunctively as a nonpharmacological distraction method for bone marrow biopsies to reduce perceived anxiety and pain in an oncology ward setting. The information gained will help to design future studies needed to ensure the efficacy and reliability of the devices.

## Methods

### Design Overview

This pilot acceptability and feasibility study was performed at the University of Florida (UF) Shands Cancer Hospital to assess VR as a relaxation and distraction tool for patients receiving a bone marrow biopsy procedure. We used a mixed methods design, in which trained research assistants identified and determined eligibility of patients admitted to the UF Health Division of Hematology & Oncology Ward in coordination with medical staff. Participants were randomly assigned to the control group and experimental group through a random number sequence to determine group allocation. No stratification was used in this process. The control (or non-VR intervention) group did not receive any special distraction in addition to local anesthesia, following conventional clinical practice for managing discomfort. Local anesthesia is defined as 5‐10 mL of lidocaine 1% (10 mg/mL) without epinephrine administered to patients for bone marrow biopsies, according to the UF Health Shands Hospital protocol. Approximately 1‐2 mL skin wheal (intradermal) was used to numb the skin, followed by a 2‐3 mL subcutaneous infiltration to numb deeper tissue, and then a 2‐5 mL periosteal infiltration for the bone surface. No additional nonpharmacological distraction methods were implemented in this group. Data regarding patient vitals, procedure length, patient feelings before and after the procedure, and patient experiences with the procedure were compared between participants assigned to the VR and control groups. The 2 groups were compared using independent *t* tests with Cohen *d* measure of effect size. For any measure taken before and after the procedure, the difference between the 2 was calculated and used to compare the groups for the *t* tests.

### Training Protocol

Research assistants underwent a 4‐ to 5-week training period covering all aspects of the patient intervention, including preprocedural preparation, informed consent, intervention administration, and postprocedural procedures. Training emphasized appropriate patient interaction, including how to explain and review consent and procedural forms. In addition, research assistants received instruction on infection control measures, such as proper sanitization of the VR equipment. These measures for the VR headsets included the use of Super Sani-Cloth Germicidal Disposable Wipes to thoroughly disinfect the headset and controllers before and after each patient’s use. In addition, research assistants were instructed to wash their hands before and after each patient interaction to minimize the risk of cross-contamination. The final 2 weeks of training focused on VR equipment operation, troubleshooting technical issues, and ensuring a smooth patient experience.

### Participants

Participants were recruited between February 2021 and February 2024 from the UF Health Oncology & Hematology ward. A total of 93 patients were initially screened for eligibility, of whom 60 patients met the inclusion criteria and were enrolled in the study, with 30 randomized to the VR group and 30 to the control group. Participants were included according to the following criteria: have a scheduled bone marrow biopsy, aged more than 18 years, and be able to physically wear and tolerate the VR headset. Participants were excluded if they had nausea or vomiting upon admission, required urgent procedures or were otherwise deemed unstable by hospital staff, had a condition that prevents the use of VR technology such as epilepsy, or a facial or scalp wound, had any visual, hearing, or cognitive impairments that would limit their ability to take part in the study, or if they could not read, speak, or write in English. The demographic characteristics of the participants are shown in Table 1. The majority of participants were male, the median age was 61.5 years (IQR 53‐68), the racial composition was 75% White with approximately 17% Black (10/60 participants) and 8% Latino (5/60 participants) enrolled, and 63% (38/60 participants) of them reported some familiarity with VR.

**Table 1.** Demographic information and familiarity with VR^a^.

Demographics	Total population (N=60)	Control (n=30)	VR (n=30)	Statistical test results for differences between control and VR groups, *t* test or chi-square (*df*)	*P* value
Age (years), median (IQR)	61.5 (53-68)	65 (58.5‐69.5)	58 (48-64)	1.31 (57.18)^b^	.92
Gender, n (%)				0.16 (1)^c^	.69
Male	36 (60)	16 (53)	19 (63)		
Female	24 (40)	14 (47)	11 (37)		
Race, n (%)				1.86(4)^c^	.76
White	45 (75)	23 (77)	22 (73)		
Black	10 (17)	4 (13)	5 (17)		
Latinx	5 (8)	3 (10)	3 (10)		
VR familiarity, n (%)	38 (63)	19 (63)	19 (63)	0.78 (1)^c^	.78

a VR: virtual reality.

b *t* test.

c Chi-square test.

### Distraction-Based VR Intervention

The “HealthPointXR” app (Gainesville, Florida), developed for this study, was used to provide distraction and relaxation for participants enrolled in the experimental group. The “HealthPointXR” app was developed by Drew Gill as part of a UF undergraduate engineering organization by the name of Dream Team Engineering. There are no financial disclosures related to the development or commercialization of this app at this time. The HealthPointXR game is currently an unlicensed, private project owned by Dream Team Engineering as per GitHub for an unlicensed project “the default copyright laws apply, meaning that [the owner] retain[s] all rights to [their] source code and no one may reproduce, distribute, or create derivative works from [their] work.” The code is currently neither open source nor free, but contacting Dream Team Engineering via their website is encouraged for any usage. This study used a retrofitted Meta Quest 2 headset (Meta) with plastic straps and easily sanitizable materials. The VR device was selected based on affordability and quality to ensure its practicality and scalability in clinical settings. The Meta Quest 2 met these criteria, while the Apple Vision Pro was considered as a potential alternative. However, due to its recent market entry and significantly higher cost, it was not incorporated into this study. The Meta Quest 2 fits most head shapes and sizes. Tablets were connected to the headsets for research assistants to control and supervise the VR content in real time using an intuitive interface. The tablet and the VR headset were connected through a Bluetooth pairing and did not require internet or Wi-Fi connectivity. “HealthPointXR” VR game included an experience of a tranquil walking path through a natural environment where the user snaps photographs of wildlife passing by while being transported in a cart along a track. The user must take these different photographs when prompted to advance their journey. Users are also incentivized to follow the track closely through minimal head movements in order to receive points on the trip across the natural environment to further immerse the user. Soothing music and different sounding notifications provide auditory cues to engage the user.

**Figure 1.**
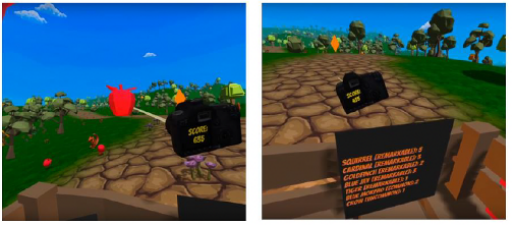
“HealthPointXR” virtual reality experience.

For participants randomly selected to the experimental group, research assistants provided a standardized 5-minute tutorial on wearing the VR headset and fitting it properly for optimal user experience. The length of time required to fit the VR headset on participants was not factored into the total length of the procedure, as it was done during normally occurring time intervals prior to the bone marrow biopsy procedure. Figure 1 shows an example of the VR experience.

### Patient Vitals and Procedure

Patients in both the VR and the control group had their vitals, including heart rate, respiration rate, and blood oxygen saturation, recorded through a pulse oximeter during and after the procedure. These vitals were taken by the health care staff present at the time of the procedure, and research assistants collected these data from the health care staff after completion of the intervention. Procedure length was recorded on a stopwatch by the research assistants, who began measuring the procedure length as soon as the VR headset was placed on the patients. Timing concluded at the end of the medical procedure once the clinician completed the last component of the procedure.

### Patient-Reported Emotional and Procedural Experience Measures

Patients in both the VR and the control group were asked the same questions regarding their feelings and experience with the procedure. Patients’ feelings regarding the procedure were assessed by pain before and after the procedure, worry before and after the procedure, anxiety before and after the procedure, and comfort after the procedure. Feelings of pain were recorded using a verbal numerical rating scale from 1 to 10 before and after the procedure with higher values indicating higher feelings of pain. Feelings of worry were measured before and after the procedure using a scale from 1 to 5, with 1 indicating “not worried at all” and 5 indicating “extremely worried.” Feelings of anxiety during the procedure were recorded on a verbal numerical rating scale from 1 to 5, with higher values indicating higher feelings of anxiety. Feelings of comfort after the procedure were evaluated using a 5-point scale from strongly disagree to strongly agree for the statement “I felt comfortable with the distraction.”

Patients’ experiences with the procedure were all assessed on a 5-point scale from strongly disagree to strongly agree. To measure awareness, patients were asked their agreement with the following statement: “I was very aware of the procedure I was receiving.” To measure distraction effectiveness, patients were asked their agreement with “Using the distraction made me feel less worried about getting the procedure.” Finally, for likelihood to repeat, patients were asked their agreement with “I would want to use the same distraction on medical procedures in the future.”

### Ethical Considerations

This study, which includes human participant research, was approved by the Institutional Review Board of the Florida Department of Health (IRB201900850). The informed consent forms, used in this study, provided participants with a description of the study, its qualitative and quantitative measures, potential risks and discomforts, and explicitly asked the patient to voluntarily agree to participate and allow their data to be collected. Study data are deidentified as all participants were assigned a numerical code. This code follows the format of OC001. Anonymity of all study subjects is ensured. No compensation of any sort was offered to human participants. This was stated in the informed consent form. No identification of individual participants is present in any images of this study or in any supplemental material.

## Results

### Patient and Procedure Summary Information

**Figure 2.**
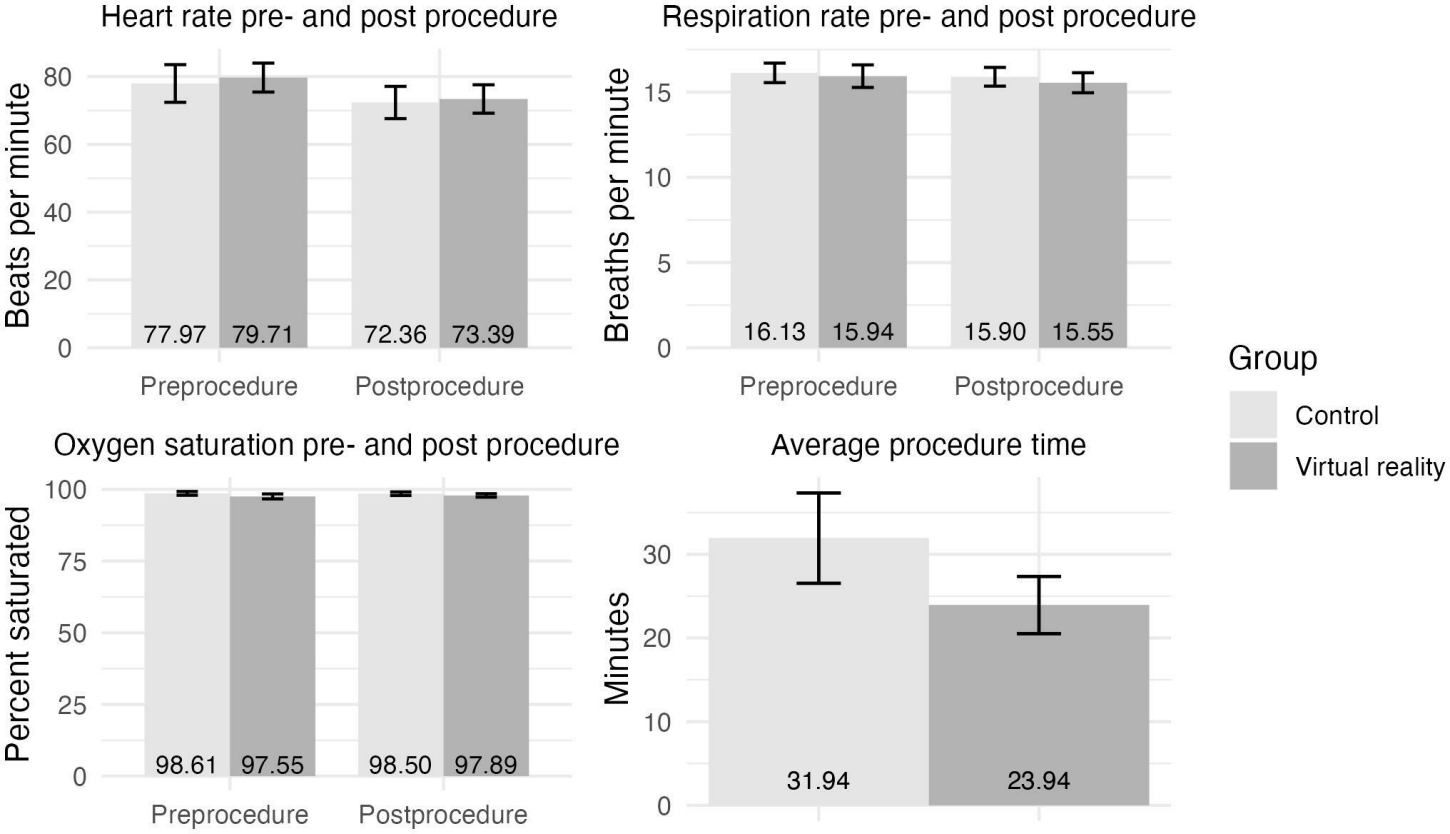
Summary of patient vitals and procedure length.

Figure 2 summarizes the patient’s vitals and procedure duration between the VR and the control group. The patient’s heart rate, respiration rate, and blood oxygen saturation were taken before and after the procedure. There were no significant differences in the change in vitals pre- and postprocedure between the VR and control group. The average procedure duration was significantly shorter in the VR group (mean 23.94, SD 9.72) compared to the control group (mean 31.94, SD 15.34; *t*_51_=−2.45, *P*=.02, *d*=0.62).

### Patient-Reported Emotional Measures

**Figure 3.**
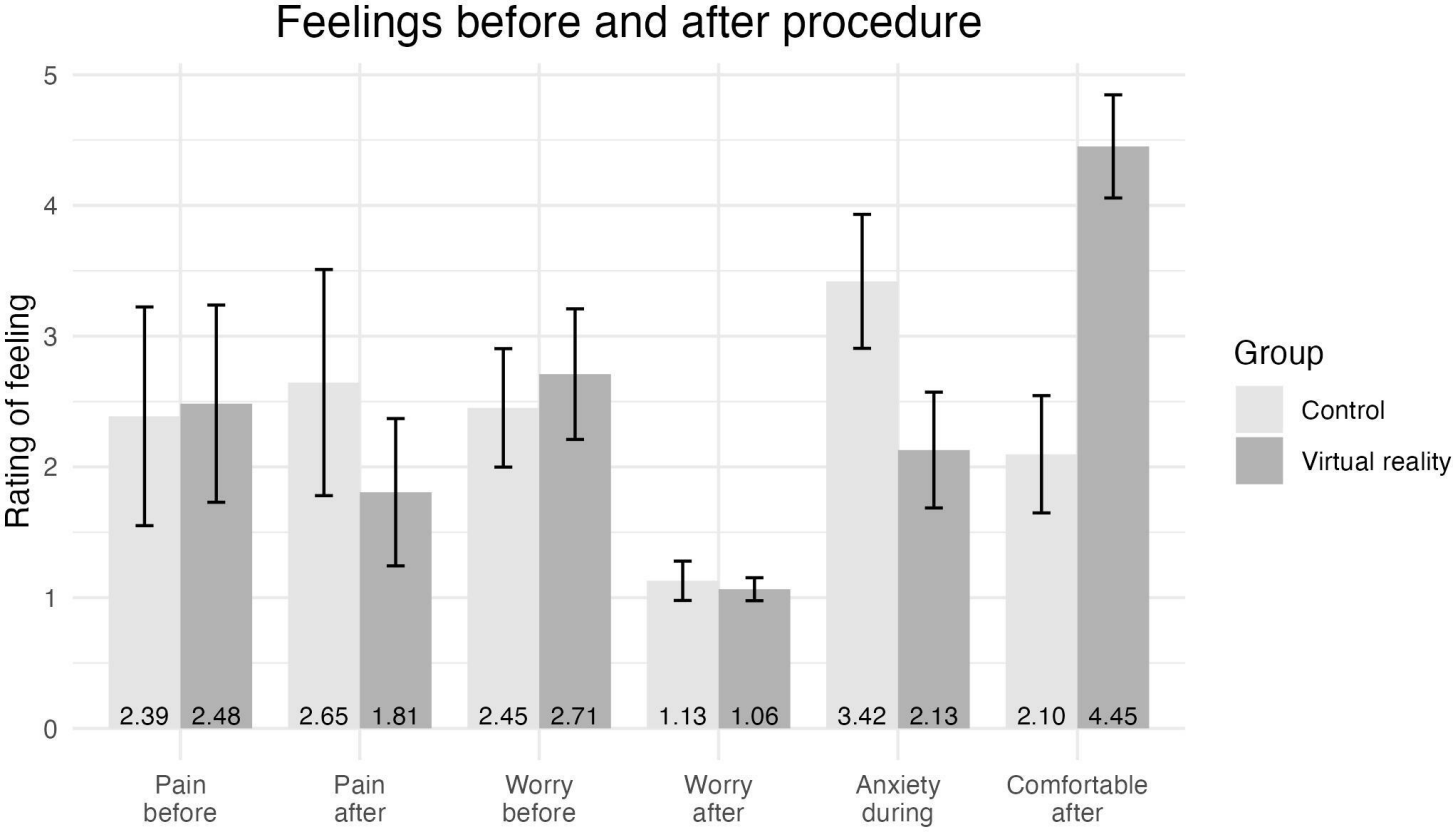
Differences in patient feelings before and after the procedure.

Figure 3 summarizes patients’ feelings before, during, and after the procedure between the VR and the control group. Patients were asked to indicate their feelings as explained in the Methods section, with higher scores indicating higher levels of pain, worry, anxiety, and comfort. The error bars represent 95% CIs, and the mean for each group is labeled at the bottom. Participants in the VR group (mean 2.13, SD 1.26) had significantly lower feelings of anxiety during the procedure compared to the control group (mean 3.42, SD 1.46; *t*_57_=−3.73, *P*<.001, *d*=0.95). Participants in the VR group (mean 4.45, SD 1.12) had significantly higher feelings of comfort with the distraction compared to the control group (mean 2.10, SD 1.27; *t*_59_=7.73, *P*<.001, *d*=1.96). There were no significant differences between the VR and control groups for pain and worry either before or after the procedure. In addition, there was no significant difference within either the VR or control group for the difference in pain or worry perception before and after the procedure.

### Patient-Reported Procedural Experience Measures

**Figure 4.**
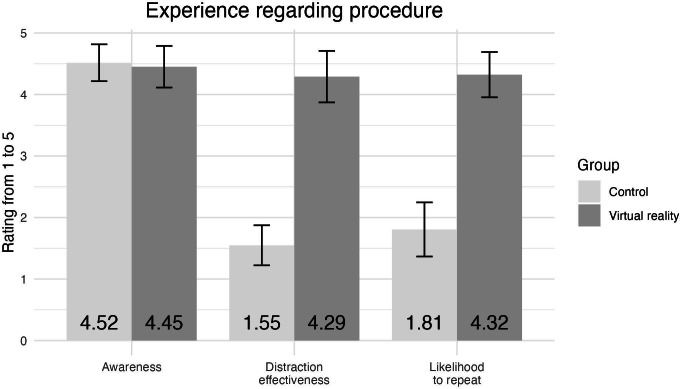
Differences in patient experience during the procedure.

Figure 4 summarizes differences in patient experience during the procedure between the VR and the control group. Patients were asked to rate the statements on a scale of 1 to 5, with higher scores indicating a larger awareness of the procedure occurring, higher effectiveness of the distraction, and a greater likelihood to repeat the procedure. The error bars represent 95% CIs and the mean for each group is labeled at the bottom. Participants in the VR group (mean 4.29, SD 1.19) were significantly more likely compared to the control group (mean 1.55, SD 0.93) to rate the distraction as effective (*t*_57_=10.14, *P*<.001, *d*=2.57). Participants in the VR group (mean 4.32, SD 1.05) were also significantly more likely compared to the control group (mean 1.81, SD 1.25) to indicate they would repeat the experience (*t*_58_=8.60, *P*<.001, *d*=2.18). However, there were no significant differences between participants in the VR group (mean 4.45, SD 0.96) and the control group (mean 4.52, SD 0.85) of how aware participants were of the procedure occurring (*t*_57_=−0.28, *P*=.78, *d*=0.07).

### Power Analysis

We conducted a post hoc power analysis using the *pwr* package in R to assess the statistical power of our independent samples *t* tests. Using the calculated effect sizes, we estimated the achieved power for each test assuming a 2-tailed *t* test with an alpha level of .05 and a sample size of 30 per group. The resulting power values ranged from 0.058 (*d*=0.07) to 1 (*d*=2.57). The estimated achieved power for 4 of the 6 independent samples *t* tests was above 0.95 (*d*≤0.95).

## Discussion

### Principal Findings

The results of this pilot study suggest that VR has a role in reducing anxiety and perceived pain in adult patients receiving bone marrow biopsy. Although this study is not designed to be statistically powered, a notable reduction in perceived pain was measured in the VR group, with a reduction of 0.7 in reported pain levels postoperatively compared to the control group’s increase of 0.2 reported pain postoperatively on a 10-point scale. These findings paralleled those reported by Wong and Choi [12] when assessing patient perceptions of VR for pain relief in labor [19].

Anxiety levels before and after bone marrow biopsies were not significantly different in the VR group compared to the control group. Anxiety in patients with cancer can be triggered by a multitude of factors, as well as anticipation of pain associated with medical procedures [20]. VR might be slightly effective in reducing reported anxiety levels, although the complex combination of factors associated with the care of patients with cancer requires caution when interpreting the results of a tool used in an acute care application. This differs from similar studies that have reported marked changes in reported anxiety levels. A study by Fabi [21] reported that among 22 patients who used VR during chemotherapy, there was a significant decrease in perceived anxiety and duration of procedure compared to the control group.

Acceptability of VR in the hospital setting was supported by the patient experience findings and reinforced by the anxiety and perceived pain data collected during this study. The results of the surveys suggest a 2‐3 point higher score in level of immersion, likelihood to use intervention again, and comfort level in the VR group compared to the control group using a 5-point scale. The majority of patients expressed a willingness to use VR as a distraction-based tool during their bone marrow biopsy. Furthermore, a study published by the *Journal of Internet Medical Research* in 2022 found VR beneficial for breaking up the monotony of treatment, providing an additional choice of activity, and in some instances a distraction from the treatment itself [22].

Measurements of feasibility for VR focused on the usability and comfort of the technology as well as its impact on the length of time for the medical procedure to be completed. To maintain methodological rigor, confounding variables were controlled as much as possible to ensure that any observed differences could be attributed primarily to the VR intervention rather than external factors. These confounding factors include standardizing the administration of lidocaine to 5‐10 mL for bone marrow biopsies within the hospital’s protocol, having the same clinician perform all the bone marrow biopsies throughout the course of this investigation, timing the procedure length to reflect the total length of the procedure as soon as a distraction method begins, that is when a VR program is turned on, before the procedure, as well as maintaining consistent informed consent and randomization practices for all participants. Consequently, the time differences observed in the results are attributed to the impact of VR. Notably, there was an 8-minute faster completion or 25% time reduction for completion of the bone marrow biopsy procedure among the VR intervention group (n=30) compared to the control group (n=30). The faster completion of the procedure occurred without any report of compromised quality of care, and these data provide a valuable clinical benchmark for the use of VR in the hospital setting, as it appears to improve the efficiency of providers while performing routine procedures. This surprising difference in procedure length is mostly attributed to the patients in the VR group being more composed and less tense at the beginning of the procedure, allowing for clinicians to more efficiently administer local anesthetic and perform the biopsy in a shorter amount of time. As the same clinician performed all of these bone marrow biopsies, clinician-to-clinician skill level was not a factor modulating the findings. The majority of the time was saved with the patient being immersed in the virtual world at the beginning of the procedure, which coincided with lower self-reported anxiety and pain ratings. It is important to note that the fitting of the VR headset on participants was performed during usual preparatory intervals prior to the biopsy procedure, and thus was not included in the recorded procedure time. The same size remains a limitation on these findings to differentiate them from being anecdotal as opposed to being powered, warranting the need for further investigation regarding time efficiencies and cost-effectiveness regarding the use of VR in minimally invasive procedures such as bone marrow biopsies.

The existing evidence to support the implementation of distraction-based VR therapy is still limited among patients with cancer receiving bone marrow biopsies [23]. This study provided insight into the novel application of a stand-alone VR headset. The results from this study indicate the use of VR as a noninferior adjunct tool that is acceptable and feasible for providing distraction and relaxation in adult patient populations undergoing bone marrow biopsy. Furthermore, these data add to building the evidence base for VR in medicine as part of innovative clinical practice involving digital therapeutics.

This study has several limitations, as the study was not designed to measure the impact of potential moderators on the outcomes. For example, the data cannot provide reliable information on the potential impact of age, gender, or pain tolerance of participants enrolled in the study. Another limitation of this study is the potential for selection bias, as participants were recruited exclusively from the UF Shands Oncology Ward in Gainesville, Florida. In addition, only patients undergoing the specific procedure of bone marrow biopsies were included, which may limit the generalizability of the findings to broader oncology populations or patients undergoing different procedures. The demographic and geographic constraints of the sample may also impact external validity, as factors such as regional health care access, socioeconomic status, and cultural background were not accounted for. The study design, sample size, and inability to double-blind the participants are also notable limitations that impacted the quality of data reported in this research. A review by Kouijzer et al [24] supports these considerations, as a lack of time and expertise on how to use VR in treatment, a lack of personalization of some VR apps to patient needs and treatment goals, or the gap in knowledge on the added value of VR in a specific setting were noted as limitations. In addition, the enrollment strategy could not control for the use of additional pain medication prior to the bone marrow biopsy that was self-prescribed by the patient prior to being approached regarding participation in the study. Furthermore, the study did not factor in medications that the patients are on or the full past medical history of participants to consider the impact of those factors on the participants’ vital signs.

### Conclusions

In conclusion, this pilot study’s findings suggest that the use of an Oculus Quest 2 Headset with the “HealthPointXR” VR program can serve as a noninferior adjunct distraction-based therapy option for patients who are distressed when undergoing a bone marrow biopsy. Based on the parameters assessed, it is reasonable to determine that the implementation of VR improved anxiety levels and perceived pain and did not prolong clinical workflow on average. Further studies with greater statistical power and a larger scale could provide more robust evidence to support the routine application of this emerging technology to impact patients’ experience during nonsedated, minimally invasive procedures.

## Abbreviations

UF: University of Florida
VR: virtual reality

## References

[1] Hyland SJ, Wetshtein AM, Grable SJ, Jackson MP (2022). Acute pain management pearls: a focused review for the hospital clinician. Healthcare (Basel).

[2] Phillips JK, Ford MA, Bonnie RJ (2017). Pain Management and the Opioid Epidemic: Balancing Societal and Individual Benefits and Risks of Prescription Opioid Use.

[3] Demir Y (2012). Pain Management-Current Issues and Opinions.

[4] Sikka N, Shu L, Ritchie B, Amdur RL, Pourmand A (2019). Virtual reality-assisted pain, anxiety, and anger management in the emergency department. Telemed J E Health.

[5] Ibitoye BM, Oyewale TM, Olubiyi KS (2019). The use of distraction as a pain management technique among nurses in a North-central city in Nigeria. Int J Afr Nurs Sci.

[6] Vambheim SM, Kyllo TM, Hegland S, Bystad M (2021). Relaxation techniques as an intervention for chronic pain: a systematic review of randomized controlled trials. Heliyon.

[7] Hu W, Yang K, Zhang L, Lu X (2021). Effect of media distraction (audio-visual and music) for pain and anxiety control in patients undergoing shock-wave lithotripsy: a systematic review and meta-analysis. Exp Ther Med.

[8] Koller D, Goldman RD (2012). Distraction techniques for children undergoing procedures: a critical review of pediatric research. J Pediatr Nurs.

[9] Bukola IM, Paula D (2017). The effectiveness of distraction as procedural pain management technique in pediatric oncology patients: a meta-analysis and systematic review. J Pain Symptom Manage.

[10] Rubio-Tamayo JL, Gertrudix Barrio M, García García F (2017). Immersive environments and virtual reality: systematic review and advances in communication, interaction and simulation. Multimodal Technol Interact.

[11] Glennon C, McElroy SF, Connelly LM (2018). Use of virtual reality to distract from pain and anxiety. Oncol Nurs Forum.

[12] Wong CL, Choi KC (2023). Effects of an immersive virtual reality intervention on pain and anxiety among pediatric patients undergoing venipuncture: a randomized clinical trial. JAMA Netw Open.

[13] Joo Y, Kim EK, Song HG, Jung H, Park H, Moon JY (2021). Effectiveness of virtual reality immersion on procedure-related pain and anxiety in outpatient pain clinic: an exploratory randomized controlled trial. Korean J Pain.

[14] Alaniz L, Sayadi L, Pakvasa M (2023). Use of virtual reality in emergency room hand procedures. Plast Reconstr Surg Glob Open.

[15] Sabinash C (2023). Utilizing virtual reality for distraction in emergency care. Curr Emerg Hosp Med Rep.

[16] Teh JJ, Pascoe DJ, Hafeji S (2024). Efficacy of virtual reality for pain relief in medical procedures: a systematic review and meta-analysis. BMC Med.

[17] Suh I, McKinney T, Siu KC (2023). Current perspective of metaverse application in medical education, research and patient care. Virtual Worlds.

[18] Smith V, Warty RR, Sursas JA (2020). The effectiveness of virtual reality in managing acute pain and anxiety for medical inpatients: systematic review. J Med Internet Res.

[19] Wong MS, Gregory KD, Spiegel BMR, Khalil C (2022). Patient perceptions of virtual reality for pain relief in labor: a qualitative study. Front Pain Res (Lausanne).

[20] Sakamoto R, Koyama A (2020). Effective therapy against severe anxiety caused by cancer: a case report and review of the literature. Cureus.

[21] Fabi A, Fotia L, Giuseppini F (2022). The immersive experience of virtual reality during chemotherapy in patients with early breast and ovarian cancers: the patient’s dream study. Front Oncol.

[22] Janssen A, Fletcher J, Keep M (2022). Experiences of patients undergoing chemotherapy with virtual reality: mixed methods feasibility study. JMIR Serious Games.

[23] Erdős S, Horváth K (2023). The impact of virtual reality (VR) on psychological and physiological variables in children receiving chemotherapy: a pilot cross-over study. Integr Cancer Ther.

[24] Kouijzer MMTE, Kip H, Bouman YHA, Kelders SM (2023). Implementation of virtual reality in healthcare: a scoping review on the implementation process of virtual reality in various healthcare settings. Implement Sci Commun.

